# Comparison of the Active Sitting Test and Head-Up Tilt Test for Diagnosis of Postural Tachycardia Syndrome in Children and Adolescents

**DOI:** 10.3389/fped.2021.691390

**Published:** 2021-09-17

**Authors:** Hong Cai, Shuo Wang, Runmei Zou, Ping Liu, Fang Li, Yuwen Wang, Cheng Wang

**Affiliations:** ^1^Department of Pediatric Cardiovasology, Children's Medical Center, The Second Xiangya Hospital, Central South University, Changsha, China; ^2^Department of Pediatrics, Xiangya Hospital, Central South University, Changsha, China

**Keywords:** postural tachycardia syndrome, head-up tilt test, active sitting test, diagnosis, children, adolescents

## Abstract

**Objectives:** We aimed to compare the hemodynamic responses to the active sitting test with the passive head-up tilt test (HUTT) in children and adolescents with postural tachycardia syndrome (POTS). We hypothesized that sitting tachycardia was also present in POTS patients during sitting.

**Materials and methods:** We tested 30 POTS patients and 31 control subjects (mean age = 12 years, range = 9–16 years) who underwent both active sitting test and HUTT successively. We measured the heart rate (HR) and blood pressure (BP) during each test.

**Results:** For both POTS patients and control subjects, the HUTT produced significantly larger HR and BP increases from 3 to 10 min of postural change than did the sitting test. Moreover, POTS patients with excessive orthostatic tachycardia during the HUTT also had significantly larger increases in HR at all test intervals during the sitting test than did the control subjects. A maximum increase in HR ≥ 22 bpm within 10 min of the sitting test was likely suggested to predict orthostatic tachycardia, yielding a sensitivity and specificity of 83.3 and 83.9%, respectively. Only six of 30 POTS patients (20%) reached the 40-bpm criterion during the sitting test, and no one complained of sitting intolerance symptoms.

**Conclusions:** We have shown that POTS patients also have sitting tachycardia when changing from a supine position to a sitting position. We believe that the active sitting test is a reasonable alternative maneuver in assessing POTS in population groups that cannot tolerate the standing test or HUTT.

## Introduction

Postural tachycardia syndrome (POTS) represents a common form of orthostatic intolerance (OI), which is not rare in children and adolescents. Approximately half of POTS patients develop symptoms before the age of 18 years (1). It can lead to physical and psychological problems, with poor quality of life. A hallmark hemodynamic criterion of POTS in children and adolescents is excessive orthostatic tachycardia: a heart rate (HR) increment ≥40 bpm or an absolute HR ≥130 bpm for children 12 years and younger or ≥125 bpm for adolescents 13–18 years within the first 10 min of the standing test or the head-up tilt test (HUTT) without a drop ≥20 mmHg in systolic blood pressure (SBP) or ≥10 mmHg in diastolic blood pressure (DBP) (2). The HUTT has been widely accepted for the evaluation of POTS in children and adolescents (3, 4).

Some patients suffering from severe OI symptoms fail to maintain an upright position or show impaired mobility (5–8). For this patient population, the HUTT may not be feasible. Tao et al. (9) described, in a study of 686 children and adolescents, that during a 10-min active sitting test, 66 children and adolescents (9.6%) reported sitting intolerance. Moreover, in that study, the sitting intolerance symptoms were related to sitting tachycardia and sitting hypertension. Sitting tachycardia was suggested with an increase in HR ≥25 bpm within 3 min after sitting. Despite these data, the differences in the physiology between active sitting and passive standing are not accounted for in pediatric POTS. Therefore, this study aimed to present and compare the hemodynamic responses to the active sitting test and the passive HUTT in children and adolescents with POTS. We hypothesized that sitting tachycardia was also present in POTS patients during sitting. The second aim of the study was to explore the optimal HR criteria for POTS diagnosis based on the active sitting test.

## Participants and Methods

### Participants

This study population consisted of 30 POTS patients (age range, 9–16 years) referred to The Second Xiangya Hospital, Central South University, and 31 age- and sex-matched healthy control subjects studied from September 2020 to May 2021. POTS patients were diagnosed using the following criteria: the presence of chronic OI symptoms (e.g., fatigue, headache, lightheadedness, blurring of vision, palpitations, tremulousness, and even syncope) and an orthostatic HR increment ≥40 bpm compared with the supine position or an absolute orthostatic HR ≥130 bpm for children 12 years and younger or ≥125 bpm for adolescents 13–18 years during the first 10 min of the HUTT, with a BP decrease <20/10 mmHg (4). Healthy control subjects did not meet the criteria for POTS. All subjects were evaluated by medical history, physical examination, complete blood count, blood biochemistry, thyroid function, electrocardiography (ECG), 24-h ECG monitoring, echocardiogram, electroencephalography, and cranium CT/MRI. Other conditions causing OI symptoms and sinus tachycardia were excluded, such as cardiac, neurologic, and metabolic diseases, recent prolonged bed rest, anemia, infection, fever, and medications (e.g., rebound effects of beta-blocker withdrawal, sympathomimetics, and anticholinergics). The Medical Ethical Committee at The Second Xiangya Hospital, Central South University, approved this research. We obtained informed consent from the guardians of the children.

### Protocol

The investigation was performed at our autonomic laboratory between 8 and 11 a.m. The environment was quiet, with stable temperature of 22–24°C. All subjects were asked to avoid taking any medication (e.g., stimulants, alpha-adrenergic receptor agonists, beta-adrenergic receptor blockers, or diuretics) that might interfere with orthostatic hemodynamic responses for more than five half-lives before evaluation (10). Also, subjects should fast for more than 4 h before testing. The testing procedure consisted of 10 min of quiet supine rest, 10 min of active sitting in a chair, followed by 10 min of supine rest, and then 10 min of the HUTT. ECG, HR, and oscillometric BP were continuously monitored. The tilting device was the SHUT-100 tilt test monitoring software system from Beijing Standley Technology Co., Ltd. (Beijing, China).

### Active Sitting Test

Subjects first lay on a tilt bed for 10 min to reach stable HR (baseline HR) and BP (baseline BP). Thereafter, the subjects were instructed to sit upright in a chair with their hands hanging down naturally, knees bent at right angles, feet on the floor, and back without any support for 10 min (9). Sitting HR and BP were recorded at 1, 3, 5, and 10 min, and then patients returned to the supine position.

### Passive HUTT

Subjects lay quietly on the tilt bed for another 10 min, with the bands fixed to prevent flexion of the ankle and knee joints, and their HR, BP, and ECG were measured again. Subsequently, the bed was tilted upward to 60° in 15 s with monitoring the HR, BP, and ECG over 10 min.

We calculated the changes in HR (ΔHR), SBP (ΔSBP), and DBP (ΔDBP) at different time intervals from the supine to the sitting or tilting posture. The maximum change in HR during the 10-min sitting test or HUTT was defined as ΔHR_max_.

### Statistical Analyses

Data analyses used SPSS 25.0 software (IBM Corp., Armonk, NY, USA) and figures were produced using GraphPad Prism 8.0 (GraphPad Software, San Diego, CA, USA). Measurement data were expressed as the mean ± standard deviation (SD) and categorical data expressed as cases (percentage). Independent Student's *t*-test or paired *t*-test or the chi-square test was used for comparisons between two groups, where appropriate. Pearson's correlation analysis was performed to examine the correlation of ΔHR between the sitting test and the HUTT. A receiver operating characteristic (ROC) curve was plotted to analyze the indicators for the prediction of the diagnosis of POTS. Area under the ROC curve (AUC) values of 0.5–0.7 indicate a “low” predictive value, 0.7–0.9 indicate a “moderate” predictive value, and above 0.9 indicate a “high” predictive value. The optimal cutoff value was determined by the maximum of the Youden index (sensitivity + specificity – 1). A value of *p* < 0.05 was taken to indicate statistical significance.

## Results

### Baseline Characteristics of the POTS and Control Groups

The study participants comprise 30 POTS patients (14 males, age = 12.7 ± 1.9 years) and 31 healthy subjects (11 males, age = 12.5 ± 1.6 years). No statistical difference was found in the age, sex, height, weight, and baseline HR, SBP, and DBP between the POTS and control groups (*p* > 0.05). However, body mass index (BMI) values in the POTS group were significantly lower than those in the control group (*p* = 0.010) (Table 1).

**Table 1:** Baseline characteristics of the postural tachycardia syndrome (POTS) and control groups.

**Items**	**Control**	**POTS**	**χ^**2**^/t value**	***P*-value**
Cases, *n*	31	30		
Male/female, *n*	11/20	14/16	0.788	0.375
Age, years	12.5 ± 1.6	12.7 ± 1.9	−0.413	0.681
Height, cm	155.9 ± 8.6	158.4 ± 8.8	−1.239	0.220
Weight, kg	49.2 ± 10.4	46.4 ± 9.3	1.086	0.282
BMI, kg/m^2^	20.1 ± 2.8	18.3 ± 2.4	2.657	0.010
Baseline HR, bpm	72.5 ± 12.2	69.3 ± 10.8	1.091	0.280
Baseline SBP, mmHg	114.2 ± 9.3	115.8 ± 11.4	−0.591	0.557
Baseline DBP, mmHg	65.7 ± 7.9	65.2 ± 8.4	−0.244	0.808

*Data are the mean ± standard deviation or number*.

*BMI, body mass index; HR, heart rate; SBP, systolic blood pressure; DBP, diastolic blood pressure*.

### Comparison of Hemodynamic Responses Between Active Sitting and Passive Tilting

**Figure 1:**
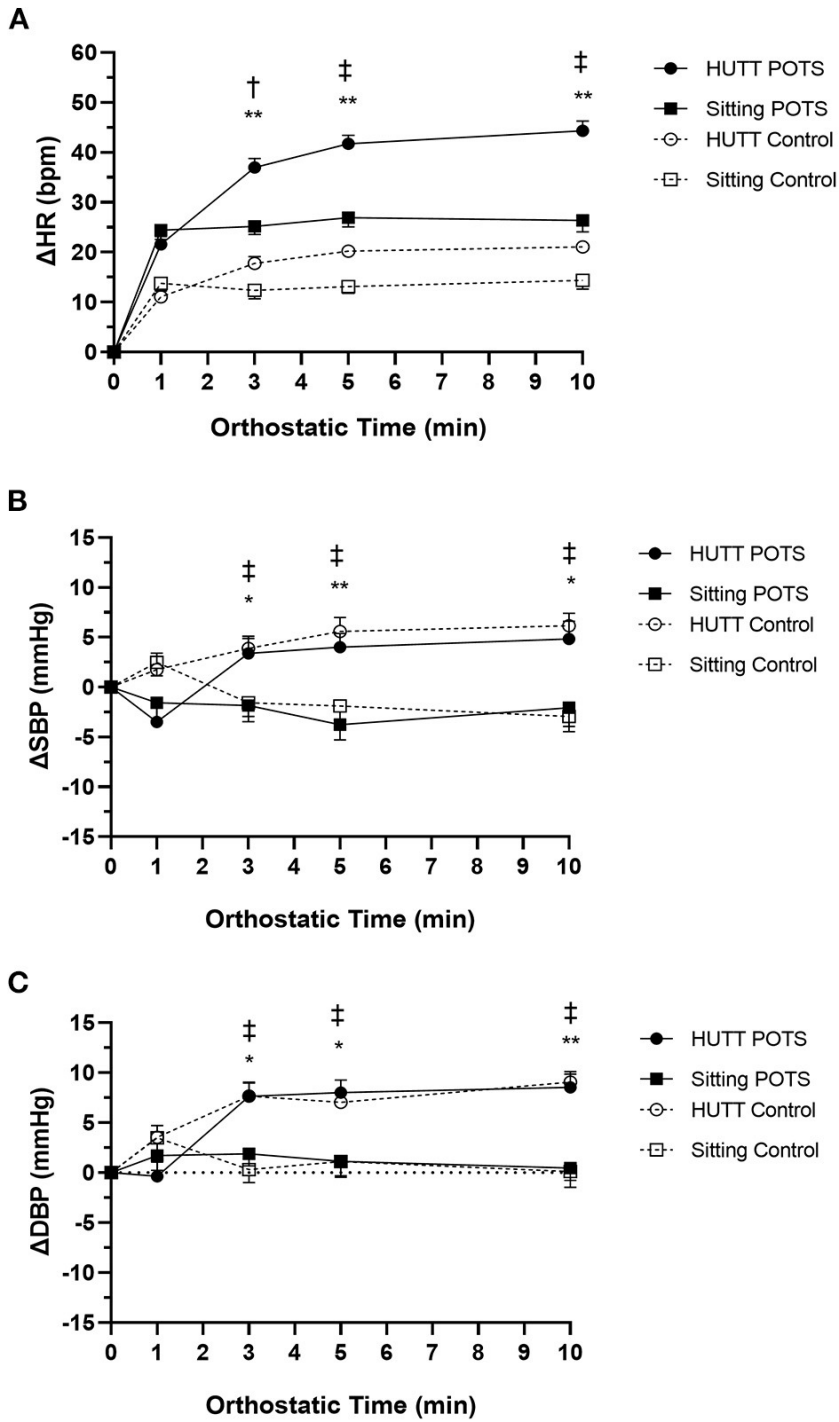
Hemodynamic responses for the postural tachycardia syndrome (POTS) and control groups during the sitting test and the head-up tilt test (HUTT). **(A)** Change in heart rate (ΔHR). **(B)** Change in systolic blood pressure (ΔSBP). **(C)** Change in diastolic blood pressure (ΔDBP) from supine (0) to sitting or tilting 10 min. Data are the mean ± standard error. *p < 0.05, **p < 0.01 indicate significant difference between the sitting test and the HUTT in the POTS group. ^†^*P* < 0.05, ^‡^*P* < 0.01 indicate significant difference between the sitting test and the HUTT in the control group.

Figure 1A and Table 2 show the changes in HR from supine to sitting or tilting for the POTS and control groups. For all participants, HR increased quickly within 1 min after sitting and tilting, then was maintained at a relatively stable level during sitting, but gradually increased during tilting. Comparison of the changes in HR between the sitting test and the HUTT with a paired *t*-test showed no significant difference in ΔHR at 1 min between the two maneuvers for both the POTS and control groups, but ΔHR was significantly greater from 3 to 10 min after tilting than that after sitting (*p* < 0.05). The ΔHR values at all time intervals during both the sitting test and HUTT were significantly larger in POTS patients than those in control subjects (*p* < 0.001). Furthermore, the ΔHR_max_ in the sitting test was significantly correlated with the ΔHR_max_ in the HUTT for all participants (*r* = 0.627, *p* < 0.001). Only 6 of the 30 POTS patients (20%) reached the 40-bpm criterion during the 10-min sitting test, and no one complained of sitting intolerance symptoms.

**Table 2:** HR responses during the sitting test and the head-up tilt test (HUTT) at different time intervals ($\bar{x}\pm S$).

**Items**	**Sitting test**	**HUTT**	***t* value**	***P*-value**
**Control**				
ΔHR_max_, bpm	16.2 ± 8.6	23.4 ± 5.9	−4.238	<0.001^*^
ΔHR 1 min, bpm	13.8 ± 8.7	11.1 ± 7.7	1.360	0.184
ΔHR 3 min, bpm	12.4 ± 9.4	17.8 ± 7.6	−2.756	0.010^*^
ΔHR 5 min, bpm	13.1 ± 7.5	20.2 ± 5.6	−4.408	<0.001^*^
ΔHR 10 min, bpm	14.4 ± 9.6	21.1 ± 6.7	−3.469	0.002^*^
**POTS**				
ΔHR_max_, bpm	30.5 ± 11.4^‡^	46.2 ± 8.9^‡^	−7.464	<0.001^*^
ΔHR 1 min, bpm	24.4 ± 10.1^‡^	21.6 ± 8.6^‡^	1.477	0.151
ΔHR 3 min, bpm	25.2 ± 8.9^‡^	37.0 ± 9.8^‡^	−6.730	<0.001^*^
ΔHR 5 min, bpm	26.9 ± 10.3^‡^	41.7 ± 9.4^‡^	−6.436	<0.001^*^
ΔHR 10 min, bpm	26.4 ± 12.8^‡^	44.3 ± 10.5^‡^	−7.523	<0.001^*^

*Data are the mean ± standard deviation*.

*HR, heart rate; ΔHR 1/3/5/10 min, change of HR from supine to sitting or tilting at 1, 3, 5, and 10 min; ΔHR_max_, maximum change of HR during the 10 min of the sitting test or the HUTT*.

* *p < 0.05 (statistically significant)*;

‡ *p < 0.001 (significantly different compared with that measured in the control group)*.

In the comparison of BP changes, for POTS patients and control subjects, the HUTT caused a significantly larger pressure increase from 3 through 10 min of postural change than did the sitting test (*p* < 0.05) (Figures 1B,C). Moreover, BP remained relatively stable from the supine to the sitting posture. No statistical difference was found in ΔSBP and ΔDBP between the POTS and control groups over time during either the sitting test or the HUTT (*p* > 0.05).

### Optimal HR Criteria Based on the Active Sitting Test for Predicting Orthostatic Tachycardia

Table 3 contains the most accurate cutoff values, sensitivities (Sn), and specificities (Sp) for predicting orthostatic tachycardia at different time intervals after sitting by ROC curve analysis. The ΔHR at 1, 3, 5, and 10 min and the ΔHR_max_ after sitting showed moderate predictive values. The optimal cutoff values of ΔHR at 1, 3, 5, and 10 min and of ΔHR_max_ were 19 bpm (AUC = 0.786, Sn = 70.0%, Sp = 74.2%), 18 bpm (AUC = 0.837, Sn = 86.7%, Sp = 71.0%), 22 bpm (AUC = 0.875, Sn = 66.7%, Sp = 87.1%), 22 bpm (AUC = 0.801, Sn = 70.0%, Sp = 87.1%), and 22 bpm (AUC = 0.867; Sn = 83.3%, Sp = 83.9%), respectively. When the cutoff value of ΔHR_max_ after sitting was set as 25 bpm, the Sn and Sp were 63.3 and 90.3%, respectively.

**Table 3:** Optimal ΔHR at different time intervals during the sitting test for prediction of orthostatic tachycardia.

	**AUC (95%CI)**	**Cutoff values**	**Sensitivity**	**Specificity**
ΔHR 1 min	0.786 (0.673–0.899)	19 bpm	70.0%	74.2%
ΔHR 3 min	0.837 (0.737–0.936)	18 bpm	86.7%	71.0%
ΔHR 5 min	0.875 (0.791–0.959)	22 bpm	66.7%	87.1%
ΔHR 10 min	0.801 (0.668–0.915)	22 bpm	70.0%	87.1%
ΔHR_max_	0.867 (0.775–0.959)	22 bpm	83.3%	83.9%

*ΔHR 1/3/5/10 min, change of HR from supine to sitting at 1, 3, 5, and 10 min; ΔHR_max_, maximum change of HR during the 10 min of the sitting test; AUC, area under curve; 95% CI, 95% confidence interval*.

## Discussion

The present study shows the different hemodynamic response profiles during the active sitting test and the passive HUTT in POTS patients and healthy children and adolescents. For both POTS patients and control subjects, the HUTT produced significantly larger HR and BP increases than did the sitting test. However, POTS patients with excessive orthostatic tachycardia during the HUTT also had an excessive increase in HR during the sitting test than did the control subjects. An increase in HR ≥22 bpm within 10 min of the sitting test was likely suggested to predict orthostatic tachycardia.

When humans assume an upright posture, the blood volume is redistributed downward by the force of gravity, with 500–1,000 ml of blood volume from the chest to the lower extremities and the splanchnic vascular bed. Prolonged standing further leads to a decrease in blood volume due to transcapillary diffusion. This redistribution reduces venous return to the heart and causes a temporary drop in arterial blood pressure. A series of compensatory reflexive responses help the body resist the effects of gravity on standing, including baroreflex, neuroendocrine responses, and “skeletal muscle pump.” These initiate a decrease in parasympathetic and an increase in sympathetic outflow, which increase the venous return, peripheral vascular resistance, and cardiac output, maintaining a relatively stable circulatory condition (11). These compensatory mechanisms cause a small increase in HR by 10–20 bpm, an insignificant change in SBP, and a slight rise in DBP by 5 mmHg (12). Failure of any compensatory mechanisms during normal standing can cause abnormal changes in HR and BP. Factors such as abnormal autonomic reflexes, hypovolemia, damaged skeletal muscle pump, a “hyperadrenergic” status, endothelial dysfunction, etc., may contribute to orthostatic tachycardia and symptoms of POTS (2, 13, 14).

The physiological responses of the passive HUTT are slightly different from those of active standing. Passive tilt allows subjects to maintain an upright posture with minimal activation of the skeletal muscle pump (10). It induces a gradual increase in HR with little or no overshoot initially. In contrast, active standing leads to a transient but a larger increase in HR during the first 30 s. There is no significant difference in the hemodynamic changes between both maneuvers during the later stage of standing (15, 16). Similarly, in our study, active sitting evoked an immediate and rapid HR increase in the initial stage. The initial HR increase on sitting may be evoked by the activation of exercise reflex and the unloading of carotid and cardiopulmonary baroreceptor reflexes with parasympathetic withdrawal (11, 15). The HUTT had a significantly larger increase in HR and BP than did the sitting test during the later stage. These reactions could be explained by the responses to the larger hydrostatic effect and the greater muscle activity in the standing position compared with those in the sitting position (17). This postural response of HR was consistent with previous studies (17–19), with an average increase in HR of around 10 bpm. Our results, which showed a negligible change in BP during the sitting test, are at variance with the previous findings (17–19). This discrepancy may be due to the different methods used to measure BP and the different age groups.

As many elderly orthostatic hypotension (OH) patients are not able to stand for several minutes, the sitting test is sometimes used as an alternative for OH diagnosis (8, 17, 19, 20). However, studies on the application of the sitting test are few in children and adolescents. Our study showed that sitting was sufficient to provoke a clinically and statistically significant increase in HR for POTS patients. Although a few patients (20%) can meet the 40-bpm criterion during the sitting test, POTS patients had a significantly larger increase in HR over time after sitting than did the control subjects. POTS patients are advised to sit or lie down when they experience OI symptoms. Our findings suggest that sitting may not be adequate enough to relieve the OI symptoms in some POTS patients.

The arbitrary limit of orthostatic increment in HR is not the only criterion for POTS diagnosis. Patients must have OI symptoms. However, the symptomatic criteria are subjective, difficult to quantify, and can often be overestimated. The change in HR is the most objective and easily measurable criterion. For some patients who are unable to stand for prolonged periods of time, a milder orthostatic stress is needed to confirm HR abnormality for diagnosis. Our findings indicate that a change in HR ≥22 bpm from a supine to a sitting position to predict orthostatic tachycardia yielded relatively favorable sensitivity and specificity, 83.3 and 83.9%, respectively. Tao et al. (9) reported that sitting tachycardia was suggested with an increase in HR ≥25 bpm within 3 min after sitting, which was related to sitting intolerance. The 25-bpm criterion in our study caused a relatively higher specificity and a relatively lower sensitivity than did the 22-bpm criterion. Moreover, in the present small study, none of the patients complained of intolerance symptoms while sitting. This suggests that this form of stress testing may be less taxing and more acceptable than the HUTT or the standing test.

There were several limitations. The number of cases in this study was not large enough, and the data were from a single center, but the main focus of our analysis is the effect of different maneuvers on ΔHR, not the disease itself. Although the study sample is heterogeneous, it can better reflect the population that will need the tested protocols. In the future, we need to carry out more studies with extensive sample size to validate the hemodynamic criteria of the sitting test for the diagnosis of POTS.

## Conclusions

This study demonstrates that a sitting test can result in a clinically significant increase in HR in patients with POTS. A change in HR ≥22 bpm when changing from a supine to a sitting position may indicate orthostatic tachycardia. Therefore, the sitting test may be considered as an alternative assessment maneuver for severe POTS patients who are reluctant or cannot tolerate the standing test or the HUTT.

## Data Availability Statement

The original contributions presented in the study are included in the article/supplementary material, further inquiries can be directed to the corresponding author/s.

## Ethics Statement

The studies involving human participants were reviewed and approved by The Medical Ethical Committee, The Second Xiangya Hospital, Central South University. Written informed consent to participate in this study was provided by the participants' legal guardian/next of kin.

## Author Contributions

HC and CW conceived the study. SW, RZ, PL, FL, and YW collected and reviewed data of the subjects. SW and RZ performed statistical analysis. HC drafted the manuscript. All authors contributed to its revision.

## Funding

This work is supported by the 2020 Hunan Province Clinical Medical Technology Innovation Guidance Project (2020SK53405, 2020SK53406).

## Conflict of Interest

The authors declare that the research was conducted in the absence of any commercial or financial relationships that could be construed as a potential conflict of interest.

## Publisher's Note

All claims expressed in this article are solely those of the authors and do not necessarily represent those of their affiliated organizations, or those of the publisher, the editors and the reviewers. Any product that may be evaluated in this article, or claim that may be made by its manufacturer, is not guaranteed or endorsed by the publisher.

## Abbreviations

AUC: area under curve
BP: blood pressure
bpm: beats/min
CI: confidence interval
DBP: diastolic blood pressure
HUTT: head-up tilt test
HR: heart rate
OI: orthostatic intolerance
OH: orthostatic hypotension
POTS: postural tachycardia syndrome
ROC: receiver operating characteristic
SBP: systolic blood pressure
Sn: sensitivities
Sp: specificities.
